# Nitric Oxide Boosts *Bemisia tabaci* Performance Through the Suppression of Jasmonic Acid Signaling Pathway in Tobacco Plants

**DOI:** 10.3389/fphys.2020.00847

**Published:** 2020-07-22

**Authors:** Yanan Xu, Cheng Qu, Xia Sun, Zhifei Jia, Ming Xue, Haipeng Zhao, Xuguo Zhou

**Affiliations:** ^1^Shandong Provincial Key Laboratory for Biology of Vegetable Diseases and Insect Pests, College of Plant Protection, Shandong Agricultural University, Tai’an, China; ^2^Department of Entomology, College of Agriculture, Food and Environment, University of Kentucky, Lexington, KY, United States

**Keywords:** *Bemisia tabaci*, nitric oxide, jasmonic acid, plant defense, tobacco

## Abstract

The intimate relationships between plants and insects start with herbivory, which can be traced back to approximately 420 million year ago. Like many other relationships, a plant–insect interaction can be mutualistic, commensalistic, or antagonistic. Within antagonistic relationships, plants deploy inducible defense to insect phytophagy. Insects, however, can evade/suppress effectual plant defenses by manipulating plant defense signaling. Previously, we showed that the sweet potato whitefly, *Bemisia tabaci*, a global invasive insect pest, can suppress jasmonic acid (JA)-dependent defenses, thereby enhancing their performance on host plants. Given that nitric oxide (NO), a multifunctional signaling molecule, interacts closely with JA signaling pathway, we hypothesized that NO is involved in the suppression of JA defensive responses. Equipped with an integrated approach, we comprehensively examined this overarching hypothesis. We showed that: (1) tobacco plants responded to *B. tabaci* infestation by accumulating high levels of NO, (2) the exogenous application of sodium nitroprusside, a NO donor, in tobacco plants attracted *B. tabaci* adults and accelerated nymphal development, whereas plants treated with 2-(4-carboxyphenyl)-4,4,5,5-tetramethylimidazoline-1-oxyl-3-oxide (cPTIO), a NO scavenger, repelled *B. tabaci* adults and prolonged nymphal development, and, more importantly, (3) silencing of *NO-associated protein 1*, a gene associated with NO accumulation, and cPTIO application disrupted the *B. tabaci-*mediated suppression of JA in plants. Collectively, these results suggest that: (1) NO signaling is activated by *B. tabaci* infestation, (2) NO is involved in the suppression of JA-dependent plant defense, and, consequently, (3) NO improves *B. tabaci* performance on host plants. Our study reflects the remarkable arm race that co-evolved for millions of years between plants and insects and offers a potential novel target (nitric oxide) for the long-term sustainable management of this global invasive pest.

## Introduction

As a result of the long-term co-evolution with insects, plants have evolved innate defensive systems to protect themselves from herbivore attacks (Feeny, 1976; Stam et al., 2014). The induced plant resistance is orchestrated by signaling networks that are directed by the phytohormones JA, SA, ETH, and NO (Scheler et al., 2013; Locato et al., 2016). The JA pathway is predominantly induced in response to wounding and tissue damage by herbivore feeding, resulting in the production of defense proteins, phenolic acids, alkaloids, or terpenoids to fend off these pests (Pieterse et al., 2012). Plants can tailor their defense to a specific attacker through accurately eliciting these signaling pathways (Reymond and Farmer, 1998). Herbivores can exploit effectors or elicitors to manipulate plant defensive responses for their own benefit by altering phytohormone biosynthesis or signaling pathways (Zarate et al., 2007; Luan et al., 2013). For instance, in the beet armyworm, *Spodoptera exigua*, feeding can directly suppress JA effectual defense and thereby enhance their feeding performance (Weech et al., 2008). Similarly, infestation by the cotton mealybug, *Phenacoccus solenopsis*, suppresses defensive responses in tomato plants through manipulating the signaling crosstalk between JA and SA (Zhang P. J. et al., 2015).

The sweet potato whitefly, *Bemisia tabaci*, Middle East-Asia Minor 1 (MEAM1), a global invasive agricultural pest, causes extensive crop damage through phloem feeding and transmitting plant viruses (Liu et al., 2007; Xue et al., 2010). To date, research on *B. tabaci* has focused primarily on inter- and intra-species competition, pesticide resistance, and reproductive interference (Mayer et al., 2002; McKenzie et al., 2002; Liang et al., 2007; Luan et al., 2012). Recently, the manipulation of inducible plant defense has received increased attention for its role in *B. tabaci* outbreaks (Tan et al., 2017; Wang et al., 2017). The competition among herbivores on the same host may rely heavily on the induced defense reactions (Kaplan and Denno, 2007). For instance, *B. tabaci* feeding can induce a specific defensive response that renders the host plant less suited for other herbivorous competitors, as observed in cabbage caterpillar, *Pieris rapae*, greenhouse whitefly, *Trialeurodes vaporariorum*, green peach aphid, *Myzus persicae*, vegetable leaf-miner, *Liriomyza trifolii*, and cotton bollworm, *Helicoverpa armigera* (Inbar et al., 1999; Zhang S. Z. et al., 2013, Zhang et al., 2014, Zhang X. et al., 2015; Zhao et al., 2019). *B. tabaci* infestation can suppress the effectual defensive response to facilitate its performance in host plants through manipulating the defense signaling crosstalk (Kempema et al., 2007). Previous studies on tomato and *Arabidopsis thaliana* have demonstrated that the JA signaling pathway is crucial in mediating induced plant defense against *B. tabaci* (Zarate et al., 2007; Zhang et al., 2013b, 2018; Shi et al., 2017). Nevertheless, *B. tabaci* feeding can suppress the induction of JA-regulated genes and defense metabolites in tobacco, *A. thaliana*, and lima beans (Kempema et al., 2007; Zhang et al., 2009; Luan et al., 2013).

Nitric oxide, a gaseous free radical, is a regulatory molecule that plays a key role in signaling plant growth and development processes, including root development, flowering, and seed dormancy (Mur et al., 2013; Scheler et al., 2013; Simontacchi et al., 2015). Although the importance of NO in plant biology has been well established, the main source of NO production is still unclear. Generally, plant NO production is mainly accomplished by enzymatic mechanisms, whereas the typical NOS protein, unlike the fully recognized NOS in mammals, has not been isolated in land plants (Jeandroz et al., 2016). However, several other pathways have been implicated in modulating plant NO levels. For instance, NR-mediated nitrate reduction contributes to NO formation (Fröhlich and Durner, 2011; Gupta et al., 2011), and a circularly permuted GTPase (cGTPase), NO-associated 1 (NOA1), has also been frequently linked to NO accumulation in plants (Asai and Yoshioka, 2009; Wünsche et al., 2011a).

The NO signaling pathway orchestrates defensive responses to a wide array of stresses, including drought, heavy metals, temperature extremes, salinity, and pathogens (Baudouin, 2011; Corpas et al., 2011; Kwan et al., 2015). Recently, NO has proved to be a multifunctional signaling molecule in plant defense against herbivore pests (Moloi et al., 2015). The involvement of NO in defense against tobacco hawkmoths, *Manduca sexta*, has been proven in tobacco plants (Wünsche et al., 2011a). In pea, NO is involved in its defense against the pea aphid, *Acyrthosiphon pisum* (Woźniak et al., 2017). Specifically, NO can affect SA/JA/ET-dependent plant defensive responses *via S*-nitrosylation of cysteine residues of the main transcription factors involved in these signaling cascades (Sun et al., 2012; Simontacchi et al., 2015; Zhou et al., 2015). For instance, NO may alter the transcription level of JA-dependent genes through changing the form of NPR1 and negatively impacting *AOC*, a key factor in JA biosynthesis, in a post-translational modification manner (Romero-Puertas et al., 2008; Caarls et al., 2015). However, the role of NO signaling in *B. tabaci*-mediated plant defensive response is still unclear.

Previous research has demonstrated that *B. tabaci* MEAM1 infestation can suppress the effectual JA defense and thus enhance whitefly performance. Because a close relationship exists between NO production and JA signaling (Huang et al., 2004; Xu et al., 2005), we hypothesize that NO is involved in the manipulation of *B. tabaci*-induced JA defensive responses. To examine this overarching hypothesis, we integrated fluorescent staining, enzyme-linked immunosorbent, virus-induced gene silencing, spectrophotometry, and quantitative real-time PCR analysis to the following questions: (1) the impact of *B. tabaci* feeding on NO signaling in tobacco plants, (2) the effect of NO on *B. tabaci* performance, and (3) the causal relationship between NO production and JA-mediated defensive pathway.

## Materials and Methods

### Effect of *B. tabaci* Infestation on NO Biosynthesis

#### Plants and Insects

Seeds of tobacco, *Nicotiana tabacum* L. variety *Xanthi-nc*, were sown in seedling-raising trays (50 cm × 25 cm) and maintained under standard greenhouse conditions: 23 ± 2°C, 75 ± 5% relative humidity (RH). When the plants were at the two- to three-leaf stage, they were transplanted into plastic pots (10 cm depth, 12 cm diameter) and placed in screened cages (insect-proof, 50 cm × 50 cm × 50 cm; 50 meshes). The plants were regularly fertilized and watered before use at the five-leaf stage.

A colony of *B. tabaci*, Middle East-Asia Minor 1 (MEAM1), was maintained on tobacco plants in the greenhouse for over 50 generations, and its identity was confirmed by a mitochondrial DNA COI marker (AY582867). All bioassays were conducted in an artificial climate chamber (RTOP-D model, Top Instrument Corporation, Zhejiang, China) under the following conditions: *L*/*D* = 12:12 h, 23 ± 2°C, 75 ± 5% RH.

#### *Bemisia tabaci* Infestation Experiments

Tobacco plants were infested with *B. tabaci* following Xue et al. (2010). Specifically, the five-leaf stage tobacco plants were placed in a screen cage (50 cm × 50 cm × 50 cm), and newly emerged whitefly adults (500 ± 10, female/male ≈1:1) were released into each cage. The whitefly adults were allowed to feed and oviposit on the plant for 24 h and were removed using an aspirator. Egg hatching and nymph development were then allowed. Plants caged without whitefly were the control plants. Leaves from infested and control plants were sampled at days 5, 10, and 15, respectively, i.e., corresponding to the 1st, 2nd, and 3rd nymph instar after the removal of adults. The fourth leaf (nine to 10 nymphs/cm^2^) was sampled for biochemical determination. Each treatment had six biological replicates per sampling date.

#### Nitric Oxide Analysis

Intracellular NO levels were detected using a method reported by Drzewiecka et al. (2014), with minor modifications. The fluorescent dye 4-amino-5-methylamino-2′,7′-difluorofluorescein diacetate (DAF-FM-DA, Beyotime, China) was used for NO level measurements. The tobacco leaves were immersed in 10 μM DAF-FM-DA solution for 30 min, washed three times with 20 mM HEPES–NaOH buffer, and mounted on a Zeiss LSM 880 inverted confocal laser scanning microscope system (Carl Zeiss, Oberkochen, Germany; emission wavelength, 515–530 nm) to estimate the fluorescence. NO content was further detected using the NO assay kit (Beyotime, China), following the user instructions. The concentration of NO was expressed in μmol/g protein. Each treatment had six biological replicates per sampling date.

#### Nitrate Reductase Activity Assay

Tobacco leaves (1 g) were ground with 10 ml of extraction buffer containing 100 mM Hepes-KOH (pH 7.5), 5 mM dithiothreitol, 1 mM EDTA, 10% (v/v) glycerol, 0.1% Triton X-100, 1 μM leupeptin, 20 μM FAD, 0.5 mM phenylmethylsulfonyl fluoride, 1% polyvinylpyrrolidone, and 5 μM Na_2_MoO_4_. The tissue homogenate was centrifuged at 12,000 *g* for 20 min, the supernatant of which was retrieved for measurement of NR activities. The NR activity was detected according to the method of Scheible et al. (1997) with modifications. To detect NR activity, one volume protein extract was added to four volumes of the prewarmed (25°C) reaction mixture containing 5 mM KNO_3_, 100 mM HEPES–NaOH (pH 7.5), and 0.25 mM NADH and incubated at 25°C for 30 min. The reaction was stopped by the addition of 0.1 M zinc acetate and was allowed to stand for 15 min, followed by centrifugation at 12,000 *g* for 15 min. After adding 1 ml of 0.02% (v/v) *N*-(1-naphthyl)-ethylenediamine in distilled water plus 1 ml of 1% (w/v) sulfanilamide in 3 M HCl, the production of nitrite was measured at 520 nm. The enzyme quantity required for catalyzing 1 μmol NO_2_^–^ within 1 h was calculated as one unit of NR. The spectrophotometric analyses were measured using UV-2700 spectrophotometer (Shimadzu, Kyoto, Japan) at room temperature. Each treatment had six biological replicates.

#### Real-Time Quantitative Reverse Transcription PCR Analysis

The transcript levels of *NOA1*, *NIA-1*, and *NIA-2* were quantified by real-time quantitative reverse transcription PCR (RT-qPCR). The tobacco plant pretreatment was performed as described above. At 15 days after the removal of the adult whiteflies, the fourth leaf (nine to 10 nymphs/cm^2^) was sampled for gene expression analysis. Total RNA was extracted using the EasyPure Plant RNA Kit (TransGen Biotechnology, Beijing, China). The first chain of cDNA was synthesized using the TransScript First-Strand cDNA Synthesis Kit (TransGen Biotechnology, Beijing, China). The RT-qPCR was carried out on a Bio-Rad CFX96 Real-Time PCR System (Bio-Rad Laboratories, Hercules, CA, United States) with SYBR-Green detection. The average threshold cycle (*C*_*t*_) was calculated per sample. The comparative 2^–ΔΔ*CT*^ method was used to calculate the relative gene expression levels. The housekeeping gene *Actin* (Genbank accession no. X69885.1) was used as an internal reference. Each gene was analyzed in triplicate in each of the three biological replicates (the sampled plants were randomly selected). RT-qPCR Primers and gene accession numbers are indicated in Supplementary Table S1.

### The Effect of NO on *B. tabaci* Performance

#### Chemical Treatment

Sodium nitroprusside or 2-(4-carboxyphenyl)- 4,4,5,5-tetramethylimidazoline-1-oxyl-3-oxide (cPTIO) was prepared in distilled water to 350 and 200 μM final concentrations, respectively, containing 0.02% (v/v) Tween 20. Tobacco seedlings with four true leaves were first pretreated with chemicals on their leaves. At 24 h after the treatment, the plants were used for the whitefly bioassay (below). Tobacco seedlings pretreated with double-distilled water served as no-chemical controls. Thereafter, the leaves of the plants were treated with SNP or cPTIO every 5 days after *B. tabaci* infestation. Each treatment had eight biological replicates.

#### Virus-Induced Gene Silencing

RNA extraction and cDNA synthesis were performed as described above. The construction of the *NOA1*-silencing vector was performed according to the method previously described by Luan et al. (2013). *NOA1* nucleotide fragments (Genbank accession no. HM755675) were amplified using target-specific primers, and the PCR product was cloned into the pBIN2mDNA1 (*Bam*HI*–Xba*I-digested) plasmid, and sequencing of the recombined vector was done to confirm the fidelity of the inserts. Electroporation was then used to transform the silencing-vector into EHA105 (*Agrobacterium tumefaciens* strain). The *A*. *tumefaciens* cultures (0.2 ml, ABS600 = 0.6) carrying pBIN2mDNA1 (*NOA1* fragment insert) were mixed with equal volumes of TbCSV (a helper virus), and the mixtures were infiltrated into the stem of each plant with four true leaves. The plants inoculated with *A*. *tumefaciens* cultures carrying pBIN2mDNA1 (without the gene insert) and TbCSV were treated as empty-vector control plants. All plants were grown and cultivated under the same conditions as described above. The second leaf from the top of the gene-silenced plants (five-true-leaf stage) was sampled for detection of the silencing efficiency using RT-qPCR. The *Actin* gene was used as an internal reference. *NOA1*-silenced and control plants were used for the whitefly bioassay (below). The primer pairs and the gene accession numbers used in this section are provided in Supplementary Table S1.

#### *Bemisia tabaci* Preference and Performance

Tobacco plant pretreatment was performed as described above (per the first paragraph of the section “The Effect of NO on *B. tabaci* Performance”). In the *B. tabaci* adult choice experiment, the treatment and the control plants were diagonally positioned in screened cages (50 cm × 50 cm × 50 cm), and 200 newly emerged adult whiteflies (female/male = 1:1) were placed into the center of the cage. After 72 h, the amount of adult whiteflies on each plant was calculated. The plants with adult whiteflies were covered in transparent plastic foil and the number of whiteflies was counted in dim light to avoid disturbance and the relocation of the whiteflies. Each treatment (one cage with two plants) was replicated six times independently.

In the *B. tabaci* adult survival experiment, 20 newly emerged adult whiteflies were released into a leaf cage (20 mm high, 80 mm diameter) attached to the stalk of the fourth leaf of chemically treated and control plants. The adult survival was consistently calculated from the average number of whiteflies per plant. Each treatment had eight biological replicates.

In the nymph *B. tabaci* development experiment, chemically treated or water-treated plants were placed in a screen cage (25 cm × 25 cm × 25 cm) (one plant per cage). About 100 newly emerged adult whiteflies (female/male ratio, approximately 1:1) were released into the cage and allowed to feed and oviposit on plants. They were removed from the plants by aspiration after 12 h of infestation and oviposition. After 20 days of adult whitefly infestation, the proportion of nymphs (first to fourth instars) was calculated on each plant to estimate the developmental rate. Each treatment had eight biological replicates.

### The Causal Relationship Between NO Production and JA-Mediated Defense Pathway

#### Quantification of Endogenous JA

Chemical treatment and *NOA1* silencing were performed as described above. Leaves from infested and control plants were sampled at 0, 5, 10, 15, and 20 days after the removal of adults. Endogenous JA content was determined using an enzyme-linked immunosorbent assay (ELISA) according to Yang et al. (2001). The antibodies used in the ELISA test were supplied by Phytohormones Research Institute (China Agricultural University, Beijing, China). In brief, 0.2 g fresh tobacco leaves was extracted for 24 h at 4°C and then purified by passing through C18 Sep-Pak cartridges (Thermo, United States). The microtitration plates were coated with 50 μl sample and 50 μl antigen (0.25 μg ml^–1^) against the JA hormones. The coated plates were then incubated for 45 min at 37°C. Next, each well was filled with 100 μl antibodies (20 μg ml^–1^) and incubated for another 1 h at 37°C. Finally, 100 μl color-appearing solution containing 2 mg ml^–1^ *O*-phenylenediamine and 0.008% (v) H_2_O_2_ was added to each well. The plates were incubated for 15 min at 37°C in the dark, and the reactions were subsequently terminated using 50 μl of 2 M H_2_SO_4_ per well. The absorbance was recorded at 490 nm. Each concentration was analyzed in triplicate in each of the three biological replicates per sampling date (each plant was sampled once).

#### JA-Related Gene Expression

Chemical treatment and *NOA1* silencing were performed as described above. At 15 days after the removal of the adult whiteflies, the fourth leaf (9–10 nymphs/cm^2^) was sampled for gene expression analysis. The transcript levels of *LOX3*, *AOC*, *TPI*, and *PI-II* were quantified by RT-qPCR. *Actin* (Genbank accession no. X69885.1) was used as the internal reference for RT-qPCR analysis. Each gene was analyzed in triplicate in each of three biologically independent treatments. The RT-qPCR primers and gene accession numbers are indicated in Supplementary Table S1.

### Statistical Analysis

Statistical significance in hormone contents, enzyme levels, gene expression, and adult whitefly performance was tested using analysis of variance (ANOVA), followed by Tukey’s test at a significance level of 5% (*P* < 0.05). The *B. tabaci* adult survival was assayed using a Cox proportional hazards model. The general linear model was used to analyze the univariate percentage of the fourth instars of *B. tabaci* on different plants. All data in the present study were analyzed using the statistical software package SPSS version 24.0 (SPSS, Chicago, United States).

## Results

### Effect of *B. tabaci* Infestation on NO Biosynthesis

**FIGURE 1:**
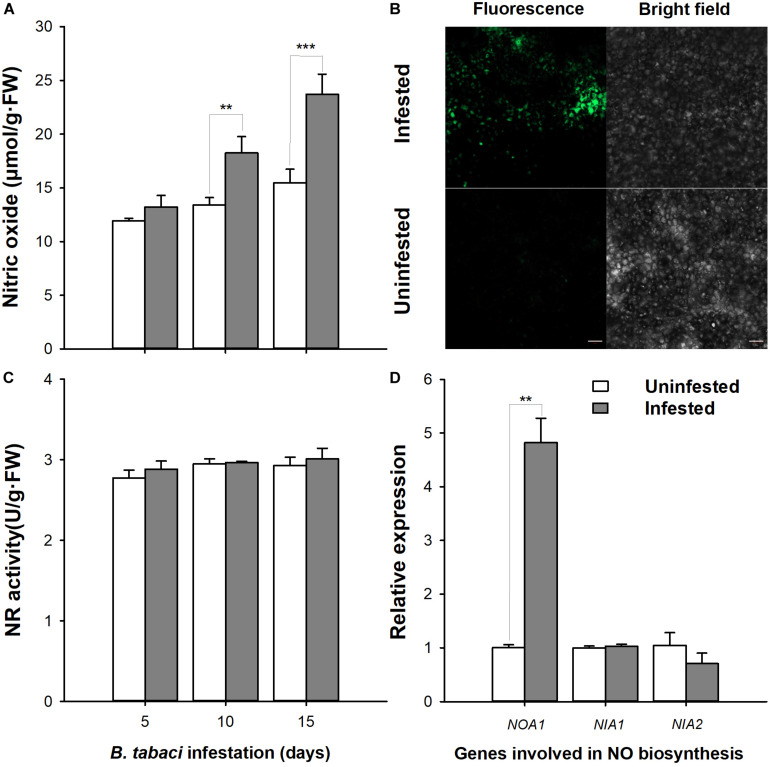
Quantification of nitric oxide (NO) levels, NO synthase activities, and gene expression in *Bemisia tabaci* nymph-infested tobacco plants. **(A)** NO production was determined by spectrophotometry after *B. tabaci* nymph infestation. **(B)** NO production was estimated using the fluorescence indicator 4-amino-5-methylamino-2′,7′-difluorofluorescein diacetate after *B. tabaci* nymph infestation. **(C)** NR activity levels in tobacco plants infested with *B. tabaci* nymphs. **(D)** *NOA1*, *NIA1*, and *NIA2* expression levels in tobacco plants infested with *B. tabaci* nymphs. Bar = 50 μm. The values are means (±E) of six biological replicates. The asterisks above the bars indicate significant differences (^∗∗^*P* < 0.05, ^∗∗∗^*P* < 0.001).

Feeding of *B. tabaci* caused a significant accumulation of NO, which was 53.41% higher than in the control at day 15 (MEAM1: *P* < 0.001; time: *P* < 0.001; interaction: *P* = 0.033, Figure 1A). Simultaneously, leaves infested with *B. tabaci* showed a greater fluorescence intensity compared with the uninfested controls (Figure 1B). However, the NR activity (MEAM1: *P* = 0.373; time: *P* = 0.260; interaction: *P* = 0.878) and transcript levels of *NIA-1* (*t* = 0.641; *df* = 3.397; *P* = 0.562) and *NIA-2* (*t* = -1.110; *df* = 3.877; *P* = 0.331) did not show obvious alterations during the entire feeding period compared with the uninfested controls (Figures 1C,D), whereas the transcript levels of *NOA1* increased, being 4.82-fold (*t* = 8.321; *df* = 8.321; *P* = 0.013) higher than that of the uninfested control at day 15 (Figure 1D). These data indicate that *B. tabaci* infestation could trigger the NO signaling pathway in tobacco plants.

### The Effect of NO on *B. tabaci* Performance

**FIGURE 2:**
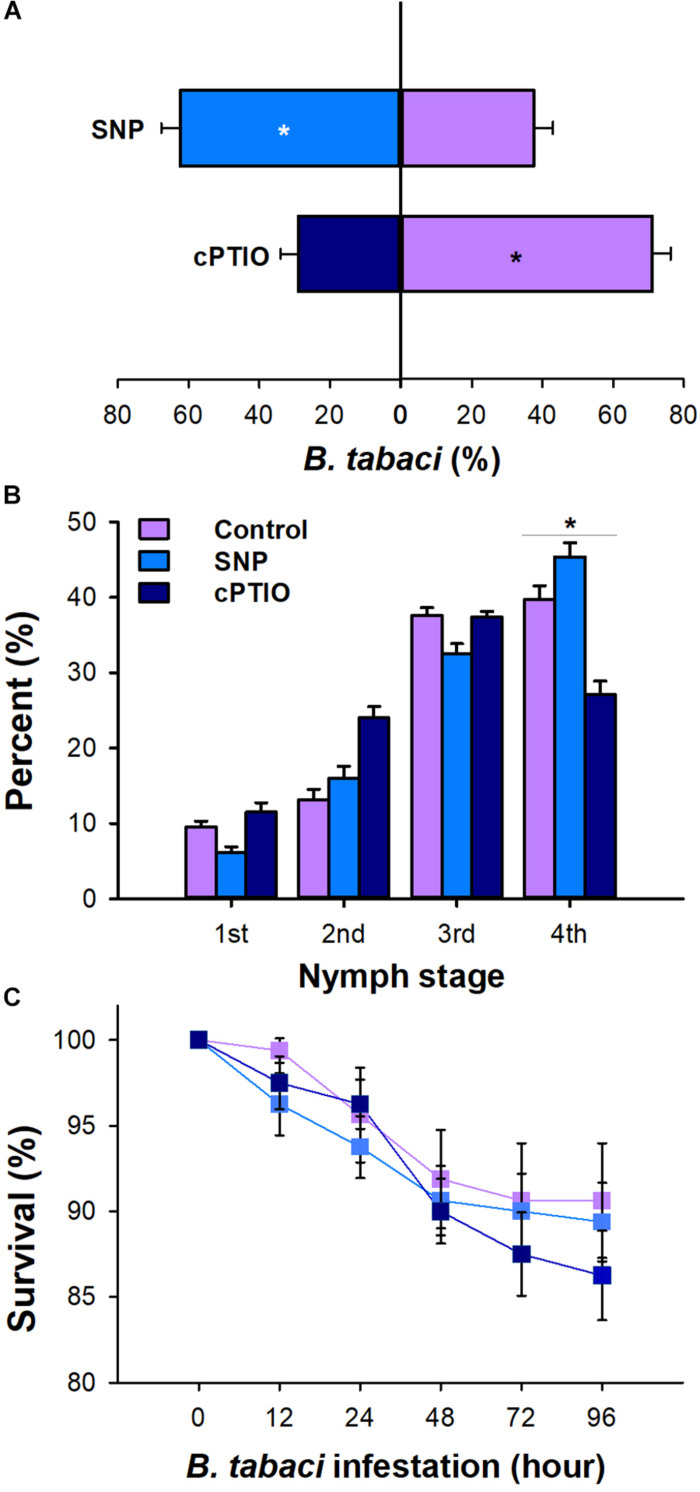
Effect of 2-(4-carboxyphenyl)- 4,4,5,5-tetramethylimidazoline-1-oxyl-3-oxide (cPTIO) and sodium nitroprusside (SNP) application on *B. tabaci* performance. **(A)** Settling preference of adult whiteflies on cPTIO- and SNP-treated plants. The values are means (±SE) of six biological replicates. **(B)** The percentage of nymph whiteflies in each instar on cPTIO- and SNP-treated plants at 20 days after the adults were removed. **(C)** Survival of adult whiteflies on cPTIO- and SNP-treated plants. The values are means (±SE) of eight biological replicates. The asterisks above the bars indicate significant differences (^∗^*P* < 0.05).

Chemical application altered the settling preference of *B. tabaci* adults. Compared with the water-treated plants, 71.09% (*P* < 0.001) of adults settled on the plants treated with SNP, whereas only 28.9% (*P* = 0.01) of adults settled on the plants applied with cPTIO (Figure 2A). Furthermore, at 20 days post-infestation, the nymph development rate differed on the SNP-, cPTIO-, and water-treated plants. The fourth instar proportion in SNP-treated plants was 14.11% higher than that in watered-treated control plants (*P* < 0.01). On the contrary, the fourth instar proportion in cPTIO-treated plants was significantly lower than that in watered-treated control plants (*P* < 0.01, Figure 2B). However, the survival rate of adult whiteflies did not differ on plants with different chemical applications (*P* > 0.05, Figure 2C).

**FIGURE 3:**
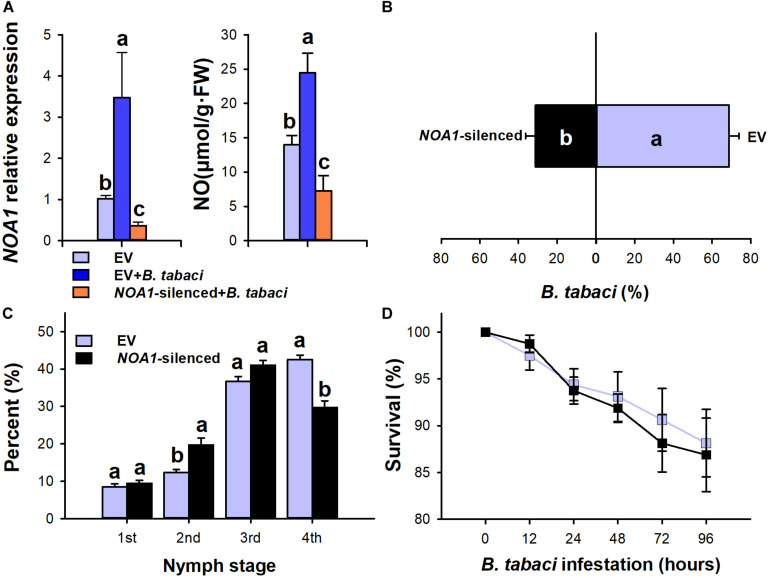
*Bemisia tabaci* performance on *NOA1*-silenced tobacco plants. **(A)** Nitric oxide content and *NOA1* transcript levels in *NOA1*-silenced tobacco plants after *B. tabaci* infestation. The values are means (±SE) of three biological replicates. **(B)** Settling preference of adult whiteflies on *NOA1*-silenced tobacco plants. The values are means (±SE) of six biological replicates. **(C)** The percentage of nymph whiteflies in each instar on *NOA1*-silenced plants at 20 days after the adults were infested. **(D)** Survival of adult whiteflies on *NOA1*-silenced tobacco plants. The values are means (±SE) of eight biological replicates. The different letters above the bars indicate values that are significantly different (*P* < 0.05).

Furthermore, the NO synthesis-related gene *NOA1* was silenced using the virus-induced gene silencing technique. As shown in the results, the transcript levels of *NOA1* and NO decreased by 0.634-fold (*F* = 6.668; *df* = 2.8; *P* = 0.005) and 51.9% (*F* = 16.256; *df* = 2.18; *P* = 0.021), respectively, in *NOA1*-silenced plants after 15 days of *B. tabaci* infestation compared with the control (Figure 3A). Similar to cPTIO application, *NOA1* silencing decreased the performance of *B. tabaci*. Compared with the empty vector-injected plants, 68.77% (*P* = 0.001) of adults settled on the *NOA1*-silenced plants, and the fourth instar proportion in the *NOA1*-silenced plants was significantly lower than that of the empty vector-injected control plants (*P* < 0.01, Figures 3B,C). However, *NOA1* silencing had no significant effect on the adult survival rate (*P* > 0.05, Figure 3D). These data indicate that a positive correlation exists between the NO level and *B. tabaci*.

### The Causal Relationship Between NO Production and JA-Mediated Defense Pathway

**FIGURE 4:**
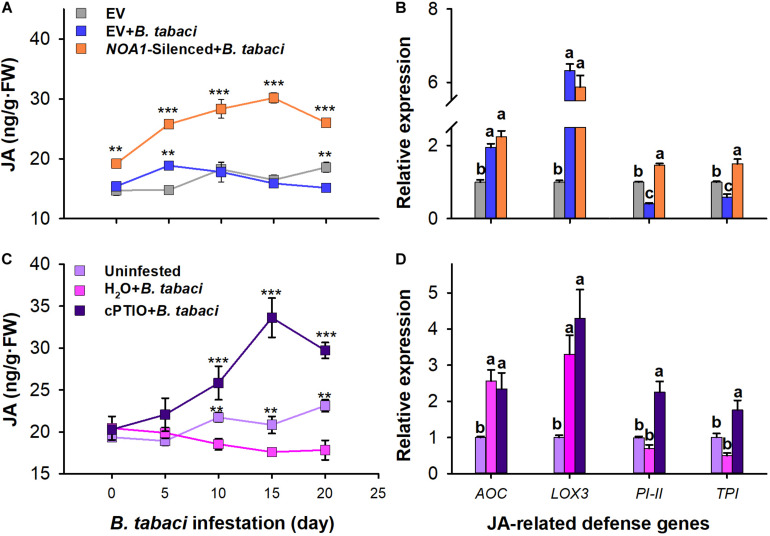
Quantification of jasmonic acid (JA) levels and expression of JA-related genes in *B. tabaci* nymph-infested tobacco plants. Jasmonic acid levels and expression of JA-related genes in *NOA1*-silenced **(A,B)** and 2-(4-carboxyphenyl)- 4,4,5,5-tetramethylimidazoline-1-oxyl-3-oxide-treated **(C,D)** tobacco plants after *B. tabaci* infestation. The values are means (±SE) of three biological replicates. The asterisks above the bars indicate significant differences (^∗∗^*P* < 0.05, ^∗∗∗^*P* < 0.001). The different letters above the bars indicate values that are significantly different (*P* < 0.05).

*Bemisia tabaci* nymph infestation has no significant effect on JA content in the leaves of water-treated plants compared with that of the uninfested controls (*P* > 0.05). However, the JA content was obviously higher in the leaves with cPTIO application than in the leaves of watered-treated controls at 0–20 days after *B. tabaci* infestation. Furthermore, the JA content peaked at 15 days, at which point it was 61.37% (*F* = 32.112; *df* = 2.26; *P* < 0.001) higher than that of the uninfested control plants (Figure 4A). In response to *B. tabaci* infestation, the variation in *AOC* (*F* = 7.309; *df* = 2.8; *P* = 0.221) and *LOX3* (*F* = 9.467; *df* = 2.8; *P* = 0.996) transcript levels did not differ between the water- and cPTIO-treated plants. However, the transcript levels of *PI-II* (*F* = 20.624; *df* = 2.8; *P* = 276 0.514) and *TPI* (*F* = 13.734; *df* = 2.8; *P* = 0.169) were not significantly affected in the water-treated plants but were increased by 2.256-fold (*F* = 20.624; *df* = 2.8; *P* = 0.007) and 1.315-fold (*F* = 13.734; *df* = 2.8; *P* < 0.05), respectively, in the cPTIO-treated plants following *B. tabaci* infestation (Figure 4B).

Similar to cPTIO application, *B. tabaci* nymph infestation obviously impacted JA accumulation in *NOA1*-silenced plants. The JA content was substantially higher in the leaves of the *B. tabaci*-infested *NOA1*-silenced plants than in the uninfested empty-vector controls at 0–20 days after infestation. Furthermore, the JA content peaked at 15 days, at which point it was 83.07% (*F* = 130.216; *df* = 2.26; *P* < 0.001) higher than that of the uninfested control plants (Figure 4C). In response to *B. tabaci* infestation, the variation in *AOC* (*F* = 34.376; *df* = 2.8; *P* = 0.221) and *LOX3* (*F* = 188.103; *df* = 2.8; *P* = 0.996) transcript levels did not differ between the empty-vector-injected and the *NOA1*-silenced plants. However, the transcript levels of *PI-II* and *TPI* were decreased by 0.408-fold (*F* = 26.966; *df* = 2.8; *P* = 0.036) and 0.591-fold (*F* = 263.591; *df* = 2.8; *P* < 0.001), respectively, in the empty vector-injected plants but were increased by 1.468-fold (*F* = 26.966; *df* = 2.8; *P* = 0.017) and 1.5-fold (*F* = 263.591; *df* = 2.8; *P* < 0.001), respectively, in the *NOA1*-silenced plants following *B. tabaci* infestation (Figure 4D). These results indicate that the application of cPTIO and *NOA1* silencing could partially alleviate the suppression of the JA-dependent defensive response mediated by *B. tabaci* infestation.

## Discussion

### *Bemisia tabaci* Infestation Can Trigger NO Signaling in Tobacco Plants

Interacting with insects for 420 million of years has led plants to evolve sophisticated defensive responses to fend off these invertebrate herbivores. *Vice versa*, insects have also evolved complex counter-defense systems for their survival, and the manipulation of plant defense signaling pathways is one of the most effective approaches (Zhang et al., 2013a). NO is one of the major plant defense signaling molecules that act in complex networks (González et al., 2012; Caarls et al., 2015). In this study, biochemical and confocal data show that NO levels were significantly increased following *B. tabaci* nymph infestation. Additionally, *NOA1* expression was consistent with that of the NO level. *NOA1* (*NO-associated protein 1*), a cGTPase, is primarily involved in ribosome assembly in plants and affects the accumulation of NO indirectly (Gas et al., 2009; Van Ree et al., 2011). These results indicated that the infestation of *B. tabaci* nymphs could trigger the NO signaling pathway in tobacco plants. Generally, NR and NOS are two major enzymatic pathways proposed for NO formation in plants (Rockel et al., 2002; Malik et al., 2011). However, in the present study, the NR activity and the *NIA-1* and *NIA-2* transcript levels were unaffected by *B. tabaci* infestation. It is now firmly evidenced that embryophytes do not possess canonical NOS (Santolini et al., 2017; Astier et al., 2018), even though putative NOS-like enzymes have been detected in some studies (Delledonne et al., 1998; Morot-Gaudry-Talarmain et al., 2002). Collectively, there is not a close relationship between NR or NOS-like enzyme and *B. tabaci*-elicited NO generation in tobacco leaves. Plants have many routes for synthesizing NO besides NR and NOS; several enzymes, including xanthine oxidoreductase, horseradish peroxidase, and catalase, are suspected to have the ability to produce NO in addition to their primary enzymatic activities (Jeandroz et al., 2016). Interestingly, the effect of *B. tabaci* infestation on NO signaling varied with feeding time. The NO-related indexes did not differ obviously from the controls until 10 days following infestation. This may be because the third nymphal instars stimulate the host plants more greatly (Estrada-Hernández et al., 2009; Wang et al., 2010).

### NO Signaling Is Positively Correlated With *B. tabaci* Performance

As a crucial signaling molecule, NO has been associated with the induced plant defensive response to phloem feeder stress. For instance, foliar treatment with NO donors induced defense reactions against the pea aphid, *A. pisum*, and the green peach aphid, *M. persicae*, in pea and sweet pepper plants (Woźniak et al., 2017; Kim et al., 2018). In our bioassay, exogenous NO donor SNP treatment significantly increased the oviposition preference of whitefly adults and accelerated the nymphal development in tobacco plants. Conversely, spraying cPTIO, a NO scavenger, on tobacco leaves rendered it more resistant to whiteflies. The performance of both adult and nymph whitefly was significantly decreased in cPTIO-treated plants. Consistent with the effect of cPTIO treatment, silencing of *NOA1* significantly decreased the production of NO, thus enhancing tobacco’s resistance to *B. tabaci*. In line with our results, *B. tabaci* performance is somewhat increased in sweet pepper after the application of NO aqueous solution (Kim et al., 2018). Wünsche et al. (2011a) found that the irNaNOA1 tobacco plants with lower NO contents are more resistant to *M. sexta* (Wünsche et al., 2011a). These results indicate that, unlike the adverse effect on phloem feeder aphids, a positive correlation exists between the NO level and whitefly performance. In addition, previous studies showed that *B. tabaci* infestation render the tobacco plants more resistant to subsequent *M. persicae* (Xue et al., 2010; Zhang X. et al., 2015; Zhang et al., 2017). Collectively, we suspected that triggering NO signaling allows *B. tabaci* to both enhance their performance and also defeat other competitors, which contribute to its outbreak.

### *Bemisia tabaci*-Induced NO Production Suppresses JA-Dependent Basal Response

It has long been demonstrated that JA is involved in plant defense to whitefly in tomato and *A. thaliana* (Zarate et al., 2007; Luan et al., 2013; Zhang et al., 2018). The present lines of evidence suggest that a close association exists between NO and JA-mediated defense (Huang et al., 2004; Mur et al., 2013). In the present research, infestation of *B. tabaci* suppressed the synthesis of JA and significantly decreased the transcript level of the downstream JA-responsive genes *TPI* and *PI-II*. This is consistent with the previous results that feeding by whiteflies suppresses downstream JA signaling defense genes in *A. thaliana* (Zhang et al., 2013b). However, cPTIO application or *NOA1* silencing alleviated the suppression of JA levels and these defense genes. In line with our findings, ir*NOA1* tobacco plants show significantly higher JA levels than wild-type plants after treatment with *M. sexta* oral secretion (Wünsche et al., 2011a). The trend of JA defensive responses was consistent with that of the whitefly performance in cPTIO-treated and *NOA1*-silenced plants. In this way, we think that the *B. tabaci*-mediated activation of NO signaling led to the suppression of effectual JA defensive responses in tobacco plants. Similarly, NO donors SNP and SNAP treatment suppresses the expression of the JA-mediated defense gene *PI* in the tomato plants (Orozco-Cárdenas and Ryan, 2002). However, in tomato plants, NO can enhance the JA-dependent defensive response against root-knot nematode (Zhou et al., 2015). This was possibly due to the high complexity of the induced plant defense reactions which may vary with the host plant species.

Nitric oxide mediated *S*-nitrosylation can affect the function of many proteins (Malik et al., 2011; Mur et al., 2013). In this study, NO-dependent post-translational modification may be a crucial explanation for the suppression of JA defense responses. In RT-qPCR analysis, *B. tabaci* infestation induced the upstream JA-responsive genes *AOC* and *LOX3* but suppressed JA synthesis and downstream JA-responsive genes. Additionally, the application of cPTIO and *NOA1* both silenced the partially alleviated *B. tabaci-*mediated suppression of JA signaling. These results are consistent with previous studies showing that while NO increases the expression of JA biosynthetic genes, including *LOX3*, *AOC3*, and *OPR3* (Mur et al., 2012; Hussain et al., 2016), the activity of the intermediate JA biosynthetic enzyme, *AOC*, can be negatively affected by NO-mediated *S*-nitrosylation (Romero-Puertas et al., 2008). Thus, we suspected that *B. tabaci* infestation suppressed the JA signal pathway in a NO-dependent post-translational modification manner. Orozco-Cárdenas and Ryan (2002) found that NO appears to be directly associated with the wounding signaling pathway downstream of the JA pathway in tomato plants through *S*-nitrosylation (Orozco-Cárdenas and Ryan, 2002). Silencing of *GSNOR1*, a nitrosylation scavenger, can enhance NO formation and decrease tobacco JA accumulation (Wünsche et al., 2011b). Furthermore, NO can act synergistically with SA-regulated responses as silencing *NOA1* can decrease the expression of *PR1*, a marker gene of the SA pathway (Asai and Yoshioka, 2009). In *Arabidopsis* and tomato plants, *B. tabaci* inhibited the expression of the JA-dependent defense gene by activating SA signaling (Zarate et al., 2007; Van der Does et al., 2013; Zhang et al., 2013b). The control of the transcriptional reprogramming of JA-induced genes by SA has been well established (Caarls et al., 2015). Recent findings have proved that NO-mediated *S*-nitrosylation can increase the affinity of cognate promoters in TGA-class transcription factors, which can bind to the *ORA59*, thereby suppressing JA-dependent defenses (Mur et al., 2013; Van der Does et al., 2013). Thus, NO may suppress JA-dependent defensive responses in a SA-dependent manner.

### Summary and Perspectives

Feeding of *B. tabaci* can manipulate the plant defense system, which constitutes one of the major factors contributing to its outbreak. NO is a crucial signaling molecule that is involved in numerous plant bioprocesses and performs sophisticated crosstalk with other plant hormones such as JA and SA. The data from this study verified our hypothesis that NO signaling is correlated with whitefly performance but is negatively correlated with JA-related defense responses. Feeding by *B. tabaci* suppressed the JA pathway and thereby enhanced its performance in a NO-dependent manner. Transcription factors can be affected by NO *via* S-nitrosylation in which NO acts as a mediator of plant defense signaling pathways. However, this study has insufficient evidence to determine that *B. tabaci* suppresses the JA defense in this post-translational modification manner. Future investigations need to explore this crosstalk at the post-translation level and to identify *S*-nitrosylated target protein in JA signaling, which will help to acquire new knowledge in MEAM1–plant interaction and provide insights for developing new methods to control this pest.

## Data Availability Statement

The datasets generated for this study can be found in the online repositories. The names of the repository/repositories and accession number(s) can be found below: 10.6084/m9.figshare.11828283.

## Author Contributions

MX and HZ designed the experiments. YX, CQ, and XS conducted the experiments. YX, CQ, ZJ, and XZ analyzed and interpreted the data. YX, HZ, and XZ drafted and revised the manuscript. All the authors read and approved the final manuscript.

## Conflict of Interest

The authors declare that the research was conducted in the absence of any commercial or financial relationships that could be construed as a potential conflict of interest.

## Abbreviations

cPTIO: 2-4-carboxyphenyl-4,4,5,5-tetramethylimidazoline-1-oxyl-3-oxide
DAF-FM-DA: 4- amino-5-methylamino-2′,7′-difluorofluorescein diacetate
DTT: dithiothreitol
ETH: ethylene
JA: jasmonic acid
NO: nitric oxide
NOS: nitric oxide synthase
NR: nitrate reductase
SA: salicylic acid
SNAP: *S*-nitroso -*N*-acetyl-penicillamine
SNP: sodium nitroprusside
TbCSV: tobacco curly shoot virus.
