# Supplementary material for: Nitric Oxide Boosts Bemisia tabaci Performance Through the Suppression of Jasmonic Acid Signaling Pathway in Tobacco Plants
Source: Front Physiol. 2020 Jul 22;11:847. doi: 10.3389/fphys.2020.00847 (PMC7387647; doi:10.3389/fphys.2020.00847)
Supplement: Supplementary file 1 [file Data_Sheet_1.docx]

**Table S1. Primers used in this study**

| **Gene** | **Accession numbers** | **Primers** |
| --- | --- | --- |
| ***RT-qPCR analysis*** | | |
| *qNOA1* | HM755675 | 5’- GCAGCCACTGCACGAAAATA -3’  5’- ACACTTTCCCACCACCAAGG -3’ |
| *qNIA-1* | JN384019 | 5’- TACACGCCTACTAGCACGATC -3’  5’- TGCCCTTTCCTTGGTATTCA -3’ |
| *qNIA-2* | X14059.1 | 5’- CTTACACGCCTACTAGCACGAT -3’  5’- TGACCTAATGGACCTTTCACG -3’ |
| *qLOX3* | AY254349.1 | 5’- GGCAGTGAAATTCAAAGTAAGAGC-3’  5’-CCCAAAATTTGAATCCACAACA-3’ |
| *qAOC* | AJ308487.1 | 5’- GAGCCAGCACCTGAAGCTAA-3’  5’- CCGGAAATGACCCCACGAAA-3’ |
| *qTPI* | DQ158200.1 | 5'-TCAGGAGATAGTAAATATGGCTGTTCA-3’  5'-ATCTGCATGTTCCACATTGCTTA-3’ |
| *qPI-Ⅱ* | Z29537 | 5’-CGTTCAGAAGGAGAGACGAT-3’  5’-TCCTGACCGAAGTAGTAGCA -3’ |
| *Actin* | X69885.1 | 5’-GCTTGCTTACATTGCTCTCGACTAT-3’  5’-GATAGAGTTGTATGTAGTCTCGTG-3’ |
| ***VIGS analysis*** | | |
| *SiNOA1* | HM755675 | 5’CG**GGATCC**TTGGTGACTGGGTTGTGGAGG-3’ (***BamH* I**)  5’GC**TCTAGA**CTTTGAGGAGCAAGAGTGGGG-3’(***Xba* I**) |

**Figure S1.** **Quantification of NO levels in cPTIO and SNP treated tobacco plants.** Different letters above the bars indicate values that are significantly different (*P* < 0.05).


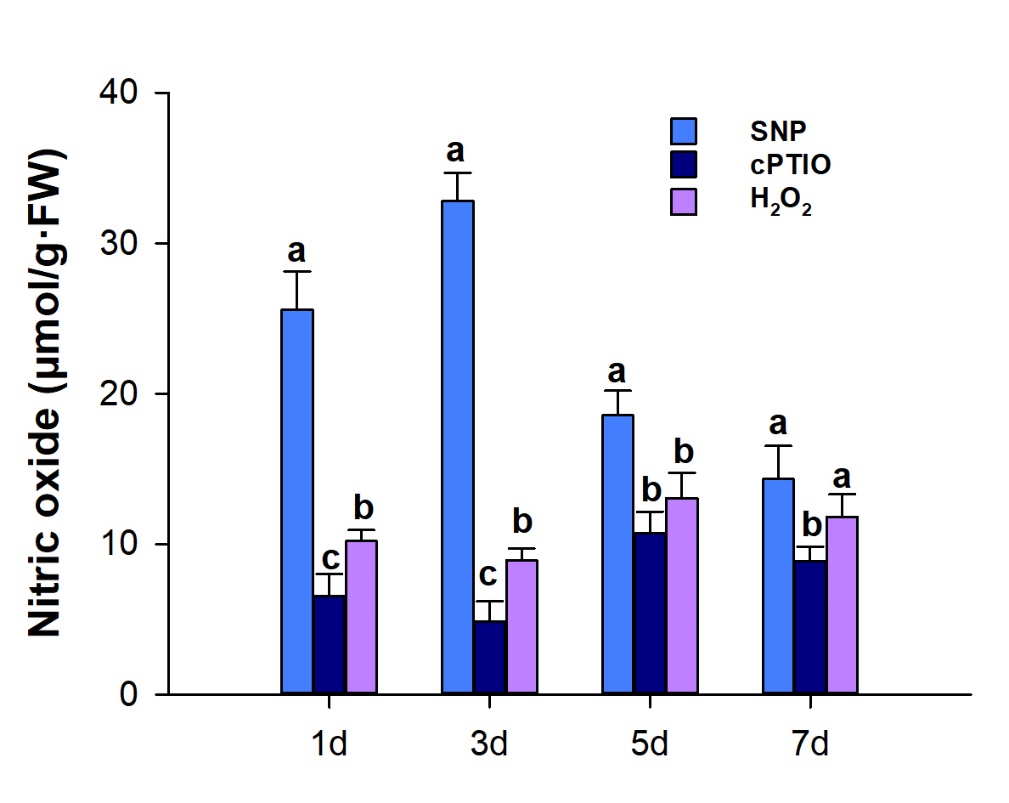


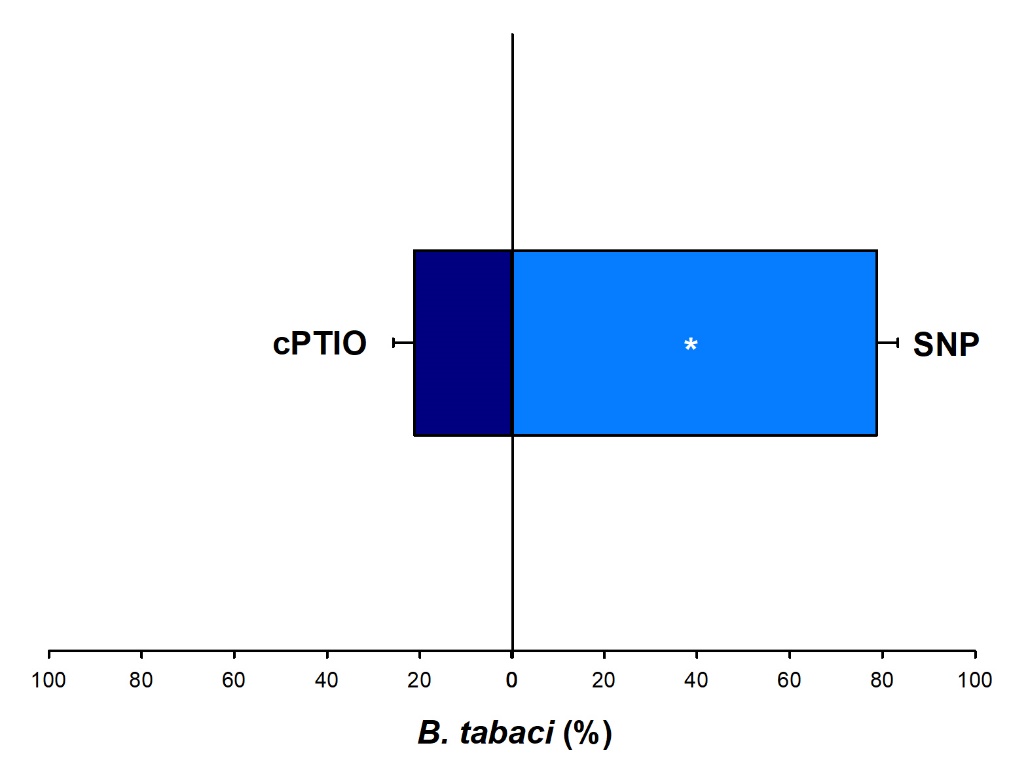


**Figure S2.** **Settling preference of adult whiteflies on cPTIO- and SNP- treated tobacco plants.** Asterisks in bars indicate significant difference.

Chemical application alters the settling preference of *B. tabaci* adults. 78.81% (*P* < 0.001) of adults settled on the plants treated with SNP, whereas only 21.18% (*P* = 0.01) of adults settled on the plants applied with cPTIO.
